# Transition Design in Latin America: Enabling Collective Learning and Change

**DOI:** 10.3389/fsoc.2021.725053

**Published:** 2021-11-03

**Authors:** Silvana Juri, Cristina Zurbriggen, Sofía Bosch Gómez, Marysol Ortega Pallanez

**Affiliations:** ^1^ School of Design, College of Fine Arts, Carnegie Mellon University, Pittsburgh, PA, United States; ^2^ South American Institute for Resilience and Sustainability Studies (SARAS), Maldonado, Uruguay

**Keywords:** sustainability transitions, societal change, transition design, knowledge integration, plurality, Latin America

## Abstract

Latin American societies currently confront numerous social, economic, and environmental issues. The complex and interlinked nature of these issues demands responses that fully and equally engage with the social and environmental domains in creative and integrative ways that blur traditional dichotomies and disciplinary boundaries. Most importantly, they need to weave in and reflect the plurality and specificity of the contexts in an autonomous and non-colonialist fashion. In this paper, we draw inspiration from approaches that emerged in the Global North such as Resilience Thinking, Policy Design, and Transition Design, all of which strive for knowledge plurality and synthesis applied to systemic transformational processes. To contribute to this pluralistic motivation and to promote critical reflection and learning, in this work we outline the main contributions of such approaches and have them converse with Latin American perspectives and practices. Through the application of a Transition Design lens, a practice-oriented perspective aimed at catalyzing societal transitional processes towards sustainable futures, we act as practitioners and interlocutors that adopt, adapt, and expand its theoretical and methodological applications in collective learning spaces, processes, and platforms. The action-oriented nature of this approach allows us to analyze particular cases of application, their contexts, and their theoretical or methodological nuances which determine their potential or degree of success in generating actual change. The structure of this article moves from outlining and introducing the main frameworks and notions relevant for adopting a Transition Design approach in Latin America, to describing cases developed in different pedagogical or action-research platforms, culminating with a collection of reflections stemming from our experiences applying Transition Design in Latin America. The first section offers a theoretical compass to expand a more robust framework that supports and enables socio-environmental transitions in the region. The second part presents three case studies to illustrate the application and interpretation of different methods and the challenges and opportunities presented. We conclude by offering insights into potential future pathways for embracing and deepening holistic and systemic approaches like Transition Design in Latin American settings.

## Introduction

Transition Design (Irwin 2015) is an emerging approach seeking to facilitate societal transition processes by supporting, connecting or developing interventions to intentionally change values, technologies, social practices, and infrastructures while reshaping interactions between socio-technical and socio-ecological systems (Ceschin and Gaziulusoy 2019). Transition Design’s (TD) tools and practices amalgamate theory and mindsets across various fields and knowledge systems, and promote collaborative spaces of practice, learning and experimentation. Its reflective and practical (Schön 1984; Steen 2013) approach to dealing with systemic issues offers a way to envision and enact alternative collective ways of being and knowing, and thoroughly embraces the concept of the pluriverse—a world where many worlds fit (De la Cadena and Blaser, 2018; Escobar 2018, 14). Its focus on deliberation, experimentation and context specificity demand that actors are encouraged to question and jointly reframe their values (Dewey 1927; Schön 1984) in a process of collective and self-transformation. Practical outcomes of this approach may include a series of material and symbolic interventions—known as “ecologies of actions”— to open opportunities that develop whole new narratives and lifestyles (Irwin et al., 2021) which unavoidably engage and challenge unsustainable values and paradigms (Du Plessis and Cole 2011).

As an emergent and fluid body of research (Irwin 2020; Irwin et al., 2021), there still exist a limited number of case studies from across the world that adopt the TD approach and its tools, although it has gained prominent traction in the past few years (Costa and Garcia i Mateu 2015; Miedes Ugarte, 2017; Hamilton 2019; Zaragoza, 2019; Owoyele and Edelman 2021). In Latin America, such examples have been scarcer, but in the past few years an increased interest has manifested in the forging of alliances between the Transition Design Institute based at Carnegie Mellon University (United States of America) and a series of educational institutions or platforms which have adopted it across the subcontinent (Zaragoza, 2019; Zurbriggen and Juri 2021; Di Bella 2022).

In an attempt to understand the features that make this approach promising to foster collective learning and systemic change around socio-environmental issues in this part of the world, we put forth a theoretical framework appropriate for this endeavor that emerges from insights on particular experiences of its implementation. We act as practitioners and interlocutors that adopt, adapt, and expand the theoretical and methodological applications of Transition Design in collective learning spaces, processes, and platforms.

## Integrating Approaches for Socio-Environmental Change

### Systemic Approaches and Their Contributions

Systemic change is complex as it spans and connects all types of subsystems—social, technological and ecological (Ahlborg et al., 2019). The interlinked nature of the social, economic, and environmental issues societies currently confront demands responses that fully and equally engage with all domains, moving beyond modern dichotomies^1^ and either/or problem framings. We adopt Resilience Thinking (Olsson et al., 2014; Folke et al., 2010) as an approach with a focus on the stability or transformation of the beneficial relations between ecosystems and society. It comes to complement the theory and tools drawn from sustainability transitions theory (Geels 2005; Loorbach et al., 2017) which focuses on the socially constructed nature of socio-technical system assemblages of norms, structures, technologies and dynamics (but excludes nature). In the recognition that changes in routines, attitudes, infrastructures, institutions and policies ensure tensions and conflicts of interest, we further adopt Policy Design (Peters 2018), a perspective that explicitly addresses the normative and political aspects that allow, mediate or prevent all transition processes. Its focus on more open, humane, systemic, anticipatory and experimental approaches (Ackoff 1974; Checkland and Scholes 1990; Rein and Schön 1994) to policymaking recognizes the key role played by the framings that determine the understanding of a problem and its potential responses. The adoption of these approaches constitutes an attempt to expand the original TD framework to integrate new theories or tools. It further proposes an attempt to re-politicize TD by explicitly considering the relevance of the restructuring of governance, power relations and empowerment while incentivizing new political capacities for transformation.

Value conflicts and disagreements are a fundamental part of any transition process (Forsyth, 2018; Bason, 2010), especially in the context of socio-environmental and socio-economic turmoil. However, the TD toolkit currently does not offer particular tools or methods to address this aspect, or to critically explore how to frame a problem. To close this gap, we propose the integration of critical systems thinking approaches (Churchman 1979; Ackoff 1974; Checkland 2000; Midgley 2000) to see systems (and their boundaries) as interpretive tools to address particular situations. Systemic Interventions (Midgley 2000) and Critical Systems Heuristics (Ulrich and Reynolds 2010) integrate the theory of Boundary Critique, a tool to analyze how to define and manage problematic situations (Churchman 1979; Ulrich 1996; Midgley 2000)—what values and purposes (and whose views) one ought to seek. A collective and normative process engaging plural and contrasting stakeholders demands addressing the politics of change. The narratives that are upholded, problematized or reshaped throughout this process determine the type of framing that is possible, as well as the type of interventions or policy instruments that may be promoted or adopted.

### Conversing With Latin-American Perspectives

While widely explored across the world, we must acknowledge that these frameworks have mostly emerged from the Global North. Situating our work in Latin America, a continent of many crises and many worlds (worldviews and knowledge systems), demands the integration of pluriversal discourses (Escobar 2020) which engage with subaltern ideas and the project of decoloniality of knowledge, very prominent in the region (Ortiz et al., 2018). This is a political and epistemic project for the emancipation of ways of knowing and being, detaching the traditionally peripheral territories from the colonial/neoliberal/modern logics. In practical terms, this means the critical reflection of the types of theories, voices and methods that are used, and the extent to which they resonate with or otherwise undermine local or non-western logics and worldviews.

As we present in our cases, notions such as *Buen Vivir, redes de solidaridad, diálogo de saberes* and *sentipensamiento,* are embedded in the ways people lead their daily activities and struggles in these territories—they nurture and influence the values that are exalted. They are both part of the vocabulary or ethos from which many participants draw from as they show up to the workshops. This suggests a demand for contextualized theories and tools that speak to the cultural nuances and specificities of the region, which will in turn shape a particular type of practice of transition design in Latin America. In the next section, we expose the main ideas and nuances that we identified in our case studies and what this suggests to future implementations in practice.

## Adopting Transition Design in Latin America: Case Studies

### Characterization of Our Cases

**FIGURE 1:**
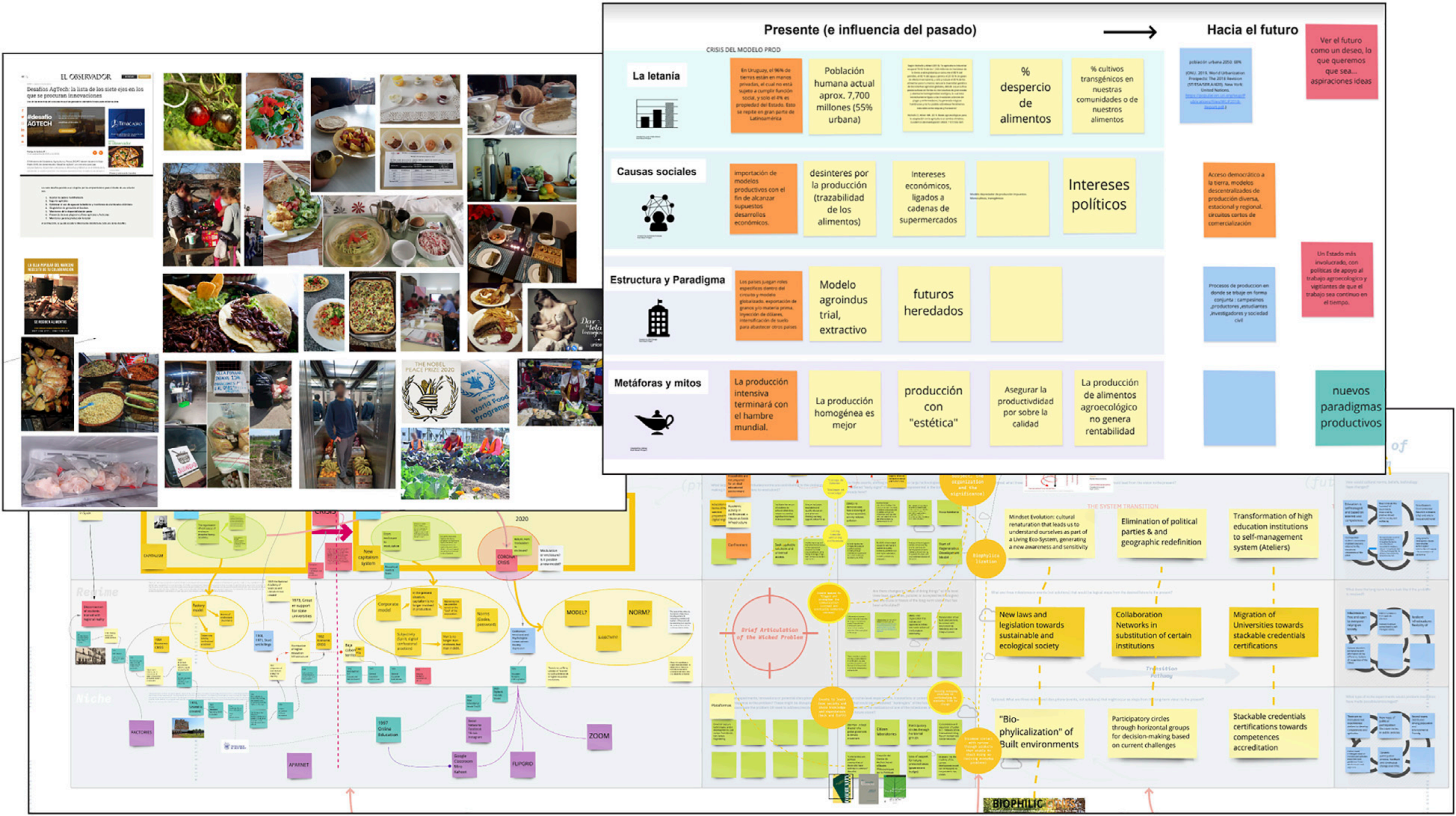
Example of boards crafted by participants, where they collaborated and made sense of the Transition Design tools and framework using Miro, the online visual collaboration tool.

We present our insights from the experiences of delivering three short courses adopting the TD approach in three contexts: Museo de Ciencias Ambientales de Guadalajara, Mexico (case 1), Instituto Tecnológico y de Estudios Superiores de Monterrey (ITESM), Mexico (case 2) and SARAS T-Lab, Uruguay (case 3). For a detailed characterization of each course, please refer to Table 1. As Latin American women, either PhD holders or PhD candidates, with strong ties to the Transition Design program at Carnegie Mellon University, we developed a comparative understanding of the cultural and social nuances that entailed delivering TD workshops in different Latin American contexts. As facilitators or assistants, we had an awareness and familiarity with the local context and idiosyncrasies, as well as the nuances that needed to be accounted for^2^. While these workshops varied in composition, length and themes, they shared various characteristics. All have been delivered online using digital tools to facilitate group activities, adopting and adapting methods that have been used previously in Transition Design projects (Figure 1). Without the erasure of the richness in diversity and nuances that make up this vast region, we find similar contextual aspects and idiosyncrasies that we recognize as features commonly present across the region—attributes that demand a differentiation of the conceptualization and practice of the still emergent TD approach.

**TABLE 1:** Overview and characterization of the three cases exposed: two training workshops in Mexico, and one in Uruguay, delivered online between July and November 2020 (during the COVID-19 pandemic).

	Case 1—MCA	Case 2—ITESM	Case 3—S T-Lab
Institution and location	Museo de Ciencias Ambientales (MCA) and Instituto Tecnológico y de Estudios Superiores de Monterrey (ITESM)—Guadalajara, Mexico	Instituto Tecnológico y de Estudios Superiores de Monterrey (ITESM) - online, catering toward 36 campuses throughout Mexico	SARAS Institute, Universidad de la República—Uruguay
	40 participants	53 participants	31 participants
Language	English and Spanish	English and Spanish	Spanish
Length and workload	6 weeks (1 session per week)	5 days (1 session per day)	8 weeks (1 session per week)
Methods and tools adopted	Fully online, canvases on MIRO	Fully online, canvases on MIRO	Fully online, canvases on MIRO
	Problem exploration through wicked problem map; Stakeholder map; Multi level perspective mapping; Narratives and visions *via* ecologies of interventions	Problem exploration through wicked problem map; Stakeholder map (simplified); Multi level perspective mapping; Visioning, milestones and backcasting; Visions *via* ecologies of interventions	Problem exploration (simplified brainstorm); Stakeholder map (simplified); Causal layered analysis; Narratives-visual collage; Vision and ecologies of interventions canvas (theory of change)
Topics covered	- Urban justice	- Racism in the Central Region of Mexico	Main theme was sustainable food and diets
	- Closing the digital gap in Guadalajara (access to internet)	- Deforestation in the Western Region of Mexico	- Food insecurity, lack of access to food
	- Sustainable transportation	- Vulnerability of the elderly in Mexico City	- Malnutrition and health
	- Sustainable co-housing	- Lack of access to public transportation in the Southern Region of Mexico	- Food sovereignty
	- Importance of museums/cultural spaces for the city	- Rural to urban migration in the North Region of Mexico	- Food culture, traditions and knowledges
	—	- The spread of and response to COVID-19 in Mexico	- Diets: processed foods and marketing
	—	- The inability of higher education Institutions in Mexico to respond quickly and effectively to Covid	—

Latin America’s multiple complex social, economic and environmental challenges are interconnected and rooted in the way transformations of technology, industry and trade have developed over time; they are also particularly linked to its colonial history (Gligo et al., 2020; Gaudin and Pareyón Noguez 2020, 25). As the most unequal region in the world (Gaudin and Pareyón Noguez 2020), many people’s daily lives revolve around the struggle to meet their most fundamental rights and needs (Max Neef, 1989), which in turn fosters multiple tensions: struggles of resistance on the one hand, and corruption and abuse of power on the other.

These contextual features had three main implications for how these workshops developed: 1) the topics, projects and challenges that participants focused on revolved around empowering individuals to lead more dignified lives; 2) environmental issues oftentimes received less attention or were perceived as less urgent (since interconnections were not immediately identified except in explicit cases related to extractivist practices or sectors (Gudynas 2011a), or due to climate disasters); and 3) the “culture of privilege” with its roots in coloniality (Gaudin and Pareyón Noguez 2020, 26) was present as a deep wound with important manifestations—i.e. coloniality of knowledge. These were also linked to profound structural issues such as: inequality and discrimination, poverty and lack of resources (funding, information) and lack of access to opportunities, which highlighted a sense of disempowerment that was often noticeable. Those who are privileged enough to have access to education often show a strong desire to familiarize themselves with novel knowledge or tools, especially if they have links to renowned global institutions. This raises a challenge for the facilitators behind these pedagogical platforms—to avoid an imposing attitude^3^ and truly integrate the richness of knowledges (*saberes*) that emerge from plural worldviews and are present in these territories.

Language is one way in which this may be expressed. Out of our three cases, only one was conducted fully in Spanish (while the other two were facilitated bilingually). This implies the potential exclusion of participants based on language-command and demanded translation efforts and the expansion of bibliography to a more contextualized one. At present, however, there is little bibliography on TD that is available in Spanish, and the theory, methods and tools that make up the TD toolkit do not circulate in languages other than English. Only in the case of SARAS T-lab, the team developed one novel activity with a mix of arts-based techniques and a prompt for a discussion suggested by one of the course participants. In all cases, the fast-paced nature of different workshops and the complex nature of the tools and activities proposed meant that participants faced challenges in fully understanding and following them. Additionally, when participants were not familiar with design methodologies, they found difficulties in generating synthesis, proposing actions (brainstorming on sticky-notes), or contributing to the virtual-board activities. The online environment represented further learning challenges that highlighted the lack of access and familiarization with the technologies used (many participants joined sessions on their mobile phones). Participants faced difficulties engaging with the multiple and foreign concepts and theory, especially in the limited time that the workshops allotted.

Another idiosyncratic feature of Latin American people highlights conviviality (Tzul Tzul 2020), an inclination towards unstructured dialogue and sharing, which presents a challenge when attempting to impose a more instrumental, fast-paced, goal-oriented focus on group activities. This prominent feature demands a radical commitment to long processes and the integration of periods of listening and observation (Bortoft, 1999 as cited in Kossoff 2011) —a true balance of action and inaction. The “optimistic grumpiness” (Tonkinwise 2015) and urgency that TD recalls, needs to be married with a culture of dialogue, listening and care, it needs to attend to our minds, bodies and hearts or what Orlando Fals Borda and others that follow him (Calderón Salazar, 2021; Escobar 2020) stress—to recognize ourselves as “feeling-thinking” beings: *seres sentipensantes* ^4^.

All in all, the three cases offered a familiarization with tools and theories, and a space to build capacities and new connections for social and transformative change. Beyond the challenges identified, participants reported to have expanded their knowledge and to have discovered a variety of concepts which challenged their more compartmentalized, non-action oriented, non-transdisciplinary or non-systemic approach to knowledge or business.

### Insights for Future Practice

Through reflection on these processes, we identified four main insights to inform future workshops:

#### Meeting Participants Where They are

As participants moved from understanding issues (framing) to identifying future pathways (re-framing), they found difficulties imagining alternative futures that would radically differ from current dominant Western paradigms. This revealed the entrenched roots related to the coloniality of knowledge and being (Ortiz, Arias, and Pedrozo 2018), and its current manifestations as inequality and shortage of opportunities. We associated this situation with the adversities of a geographic region in pain. Every day struggles to meet basic human needs (Max Neef 1989) make worlds crumble (Scarry 1985) and precludes a free imaginative roaming into beyond than presently possible prospects. When participants adopted other-than-western worldviews, there was a direct connection to the indigenous notion of *Buen Vivir,* a fluid concept adopted in the region to refer to multidimensional and socio-environmental wellbeing (Gudynas 2011a). Meeting participants where they are, with an understanding of their own positionality becomes pivotal for approaches that aim to connect the local with the global and encourage action at different levels of scale. A longer timeframe for the workshops (meaning also a slower pace) can encourage participants and facilitators to explore and select their own working tools, adapt already existing ones and innovate in the way they utilize and deploy them.

#### A Risk of Echo Chambers

All three cases were disciplinarily diverse, yet most participants had connections with academic or research institutions. We hypothesize that this homogeneity can significantly shift the experience and results of the workshops. In order to avoid echo chambers, the integration of diverse and contrasting views is decisive. However, this raises the questions: what would an engagement of radically divergent voices entail? What sort of care and negotiation strategies would be required from organizers and facilitators to manage a variance of power dynamics? The current toolkit does not allow to exercise this balancing of interests and deep deliberation, and has yet to create space to develop the required tools and skills. Insights from critical systems thinking seem to suggest new strategies and tools to explore.

#### The Need for Stated Values

Transition Design has been developed as an approach where a variety of methodologies and methods could be explored and adapted to fit local circumstances. Being a values-driven approach, the values of the locality approaching a complex issue will be determinant in the practical application of TD. The dynamism of values then begs the question: what are the values rooted in a perspective of Transition Design that stem from an anglo-european perspective and what would the values be in a Latin American context? The mundane, the commons, the relationship with what is considered public and indigenous is inherently different in each context, and often regionally divergent and contrasting. A positionality with a clear statement of the overarching values from the working team and participants seems like a precondition for a suitable landing of the approach and its fit with the particular place.

#### An Orientation to Collaboration for Action

The main outcomes of the three cases present these spaces as an initiation into systemic-change processes and the beginning of dialogues and collaboration in regard to multiple thorny subjects. A conclusion of the workshop processes is that they can be understood as introductory platforms that foster synergies for activist practices. The reactions to current adversities, aim to shift current *problematic situations* (Checkland 2000; Reynolds and Holwell 2010; Sydelko et al., 2021) into preferred circumstances (Irwin 2015), and makes this approach one oriented towards action rather than knowledge production. Indeed, while bibliographic materials were offered throughout in all cases, a lack of engagement confirmed this is not where the main motivations or needs resided. We therefore envision the evolution of TD, especially in Latin America, towards the incorporation of building continuous and “value-full” (Lewin, cited by Midgley, 2000) practices, balancing actions and inactions over time, with time for observation and dialogue. This should foster more cross-pollination and learning between and from participants.

## Discussion

### Supporting Pluriversal Futures

One of the pressing challenges the Transition Design framework poses in its adoption in Latin America is to incur or reproduce colonial ideologies and fail to support *pluriversality* (Lander and Castro-Gómez, 2000"). Transition Design processes and tangible actions need to be constantly supporting and learning from the communities that are shaping and benefiting from those. To ignite societal changes for futures worth living, the designing for transitions from place, by place, and for place should incorporate ways of sensing, thinking, making and unmaking futures that are native to the place. It must resonate with localized cultures and languages to avoid the erasure of their ethos. This means being sensitive to the consequences and legacy of colonialism and its present-day manifestations as colonialities of thinking and being (Ortiz et al., 2018, 34).

This emancipatory endeavor implies the avoidance of the reproduction of ontologies and ideologies from western ideas of development and growth. As Escobar (2020) and others (Kothari et al., 2019) stress, we need to think of alternatives *to* development since this concept is deeply entrenched in a modern (and thus colonialist/cartesian) worldview that does not take into account planetary boundaries and currently puts Earth systems and societies at risk (Gudynas 2011a; Raworth 2017; Escobar 2018; Ceschin and Gaziulusoy 2019). Alternative platforms and frameworks are necessary, to align with the plurality of views, understandings and ways of being that exist in Latin America today cohabiting territories in harmony with the natural world. This means a revitalization and revaluation of indigenous worldviews and their wisdoms (practical, political and ethical) without leaving behind the role that science and technology have to play.

The umbrella concept of *Buen Vivir* (Gudynas 2011b) is an exemplar of a political and ethical platform that encompasses plural understandings of a good life (multidimensional well-being), only possible in a community in which all beings (including humans) coexist, constitute, and sustain each other in solidarity. *Buen Vivir*, as a fluid concept, represents a critique of development rooted in “conventional Eurocentric knowledge” (Gudynas 2011b), and a decolonial endeavor that allows the expression of different ontologies with their unique understandings and feelings of the world (Gudynas 2011b). The socio-ecological conception of community and wellbeing of all Earth-beings inherent in the notion of *Buen Vivir*—with similarities to Resilience Thinking (Folke 2016), constitutes a fundamental concept to adopt, explore and converse with the multiple cultural identities that may be expressed or prefigured through Transition Design in Latin America.

To complement this notion, we also find *redes de solidaridad* (networks of solidarity) (Marín-Herrera et al., 2001; Hernández de Padrón, 2006; Giglia 2014; Mata-Marin 2020), a type of bounding practices compound by invisible and visible tactics (Mata-Marin 2020). These strategies or tactics stem from a “logic of solidarity,” where the care for collective wellbeing materializes through invisible “strategies to bypass structural barriers in an effort to improve (people’s) living conditions and fulfill everyday life needs,” and visibly “(exert) organized political action intended to potentially generate change” (Mata-Marin 2020, 143). The interplay of acting invisibly to change the present and acting visibly in the present to change the future becomes a form of future-making that transcends current conceptions or TD tools used for visioning and backcasting (Irwin 2020).

Finally, these notions remind us of the concept of *diálogo de saberes* (dialogue of knowledges) (Delgado 2016), prominently adopted in Latin America as the integration and dialogue of western science with local sciences and traditional or indigenous wisdoms. This “dialogue” implies holding a space-time to talk and “do science with the heart”—it expresses what is of value to us, our “con-science” (Betancourt Posada and Gómez Cruz, 2019, 149). *Diálogo de saberes* is a true expression of knowledge integration and transdisciplinary, but not from a romantic view of the past. Instead it makes our ancestral wisdom into a warp to weave in desirable futures that honor the biocultural diversity of place, a Latin American futurity that incorporates indigenous social thinking (Garzón 2020). This allows us to reinvent futures that emanate from ancestrality (Red Temática de Patrimonio Biocultural México 2016). From this perspective, TD in Latin America could emerge as a practice of cultural, narrative and aesthetic “re-existence” (Albán-Achinte 2012)—a critical and pragmatic approach to envision and “filter the future”^5^ (Betancourt Posada and Gómez Cruz, 2019, 105) that may allow the possibility to enact pathways to sustainable and plural futures.

### Challenges and Opportunities Presented

As practitioners attempt to synthesize and integrate knowledge and tools emerged in anglo-european contexts with the particularities of other territories, this process demands to acknowledge positionality and explicitly adopt the insights from critical systems approaches (Reynolds and Holwell 2010, 10). In particular, it prompts us to see TD as a process aiming at developing “systemic interventions”, performing purposeful actions by agents “to create change in relation to reflection on boundaries” (Midgley, 2000, 132). The definition of such boundaries—from the adoption of boundary critique (Ulrich and Reynolds 2010)—can help articulate the values and goals that are present not just in the participants or stakeholders involved, but also on the side of researchers or facilitators. Following Midgley (2000, 130), the very adoption of theories and methods are a form of action and as such should be scrutinized to unconceal hidden assumptions, reveal interests and potential power tensions or conflicts.

As we have seen in our cases, the design of these pedagogical spaces demands an openness to a multiplicity of worldviews with their knowledges and tools, which are unavoidably driven by different purposes. Thus, this demands “the creative design of methods” (Midgley, 2000, 226), a synergistic combination of tools that address distinctive questions within the issues of concern. This process of adoption and adaptation finally constitutes a whole that is more than a mere sum of its parts, and differentiates itself from an otherwise linear or formulaic adoption of rigorous and external methods. The flexibility in this creative adaptation eases and supports a more appropriate and contextual specificity in the process—with particular purposes, needs and capacities that demand to be leveraged. The outcome of such an open, fluid and reflective process can therefore emerge as a mutual learning space and process which encompasses the exploration and integration of useful knowledge—either tacit or codified—for a deeper understanding of a problem, better decision-making and, therefore, transformation and change (Westberg and Polk 2016).

## Closing Remarks

Transition processes imply transformational learning and change, with the participation of stakeholders with diverse and often divergent types of knowledges, interests and worldviews. Creating the conditions for enabling sustainable futures requires radical, systemic changes in values, beliefs and in patterns of social behavior (Westley and Laban, 2015) which in turn require an ontological-epistemological revolution that can truly re-embed the social spheres within nature—understanding its interdependencies.

This work attempts to outline a framework that adopts and adapts the Transition Design approach to educational institutions and platforms in the context of Latin America. Through the critical engagement with different systemic approaches and the analysis of three cases, we put forth a theoretical topography of what Transition Design workshops, or alternative pedagogical spaces, would need to engage with, integrate or expand within the region. We identify that the rich and diverse context that constitutes this subcontinent presents a series of challenges and opportunities for the adoption of TD as a type of systemic intervention that aims to ensure pluriversality, solidarity and mutual learning (transdisciplinarity).

In an attempt to initially characterize TD in Latin America, we draw the conclusion that it constitutes a dialogical empowering platform for activist practices fostering socio-environmental transformations through synergistic collective actions that constitute *systemic interventions*. Such spaces, while motivated from a sense of urgency, a pressing need for transcending inequality and enabling dignified lives, need to harmonize with the pace, aesthetics and ethics of life in the region, as we see with endeavors as Indigenous Futures (Futuros Indígenas 2021). Conviviality, solidarity, a horizontal exchange and valuation of knowledge and wisdoms, the explicitation of values and an inclination to caring practices of re-existence towards *Buen Vivir* (acting with the heart using the head in communion with nature) are its most outstanding features.

Lastly, the relevance of this analysis could transcend the initial regional focus. While this paper focuses on the identification of key contributions or notions present in the Latin American territories, these theoretical and practical considerations to allow emancipatory collective learning processes for action, could also be valid to steer the exploration of similar perspectives in other regions. Hence, this paper becomes an invitation for further research to explore the development and implementation of this framework and its associated tools in-practice.

## Data Availability

The original contributions presented in the study are included in the article/Supplementary Material, further inquiries can be directed to the corresponding author.

## Footnotes

1 Following Latour’s (2005) conceptualization of modernity as a process that attempts at dichotomous bifurcations: natural science from social science, science from politics, nature from culture and intuition from reasoning (also Scheffer et al., 2015).

2 Nevertheless, we understand our own positionaly as interlocutors in these spaces and therefore the following reflections stem from our perspectives during the workshops and would need further validation from participants

3 One of the critiques of imposing attitudes can be equated to a process of colonization, “colonization of knowledge and of being” as explained in Ortiz et al., 2018. The power dynamics of researchers, practitioners and facilitators are always challenged or questioned, so a self-critical attitude is deemed key (Coghlan and Shani 2005).

4
*Sentipensar*, a term originally reported by Orlando Fals-Borda in 1984, means “acting with the heart using the head”, as used by people of the Caribbean coastal region, also popularized by Eduardo Galeano and Arturo Escobar (Botero Gómez in Kothari et al., 2019, 304).

5 A concept reported by Víctor Manuel Toledo and used by members of the peasant Tosepan cooperative in Puebla, Mexico. It refers to the domestication of science and technology, as an adaptation to the local conditions and interests–a type of innovation that is communally directed and that places science in an important yet not fallible place. The community filters the future by learning and adopting science but also as it is left to “decant” (p.108).
